# The incidence of hepatocellular carcinoma and clearance of hepatitis B surface for CHB patients in the indeterminate phase: a systematic review and meta-analysis

**DOI:** 10.3389/fcimb.2023.1226755

**Published:** 2023-09-12

**Authors:** Min Liu, Taixue Zhao, Yuting Zhang, A-Mei Zhang, Jiawei Geng, Xueshan Xia

**Affiliations:** ^1^Faculty of Life Science and Technology, Kunming University of Science and Technology, Kunming, Yunnan, China; ^2^Department of Infectious Disease and Hepatic Disease, The First People’s Hospital of Yunnan Province, Kunming, China; ^3^Medical School of Kunming University of Science and Technology, Kunming, China; ^4^Kunming Medical University, Kunming, China

**Keywords:** chronic hepatitis B, indeterminate phase, HBsAg, clearance, HCC, meta-analysis

## Abstract

**Background:**

Nearly 30%–40% of patients with chronic hepatitis B do not fall into any of the traditional natural history classification and thus are classified as indeterminate. However, it is unclear whether patients in the indeterminate phase (IP) are at a higher risk for hepatocellular carcinoma (HCC) than those in the defined phases (DP) and would benefit from antiviral therapy. We performed a systematic review and meta-analysis of HCC incidence and HBsAg clearance among patients in the IP versus DP.

**Methods:**

We defined the clinical phases as per the AASLD 2018 hepatitis B guidance. We searched PubMed, Embase, Medline, and Web of Science for relevant studies that reported HCC incidence or HBsAg clearance in IP versus DP patients published between January 2007 and March 2023. Annual HCC incidence and HBsAg clearance rates were pooled using a random/common-effects model.

**Results:**

We analyzed data from 14 studies, comprising 7798 IP patients (222 patients developed HCC and 239 achieved HBsAg clearance) and 10,725 DP patients. The pooled annual HCC incidence was 2.54 cases per 1,000 person-years (95% CI, 1.14–4.39) and HBsAg clearance rate was 12.36 cases per 1,000 person-years (95% CI, 10.70–14.13) for the IP patients. IP patients were associated with significantly higher HCC incidence risk (RR = 1.64, 95% CI, 1.34–2.00) and slightly lower annual HBsAg clearance rate (RR = 0.83, 95% CI, 0.70–0.99) than the DP patients. In addition, HBeAg-negative IP patients (2.31%; 95% CI, 0.87–4.45) showed a significantly higher HCC incidence than those who were HBeAg positive (0.00%; 95% CI, 0.00–0.99) (*p*< 0.001). The Asia-Pacific region IP patients (4.30%; 95% CI, 2.07–7.27) were also associated with a higher HCC incidence versus Europe (0.05%; 95% CI, 0.00–1.39) (*p*< 0.001). However, there were no significant differences between different strategies (treated vs. untreated: 2.56%; 95% CI, 1.01–4.63 vs. 1.61%; 95% CI, 0.00–5.81, *p* = 0.09), and heterogeneity was substantial across the studies (*I*^2 =^ 89%).

**Conclusion:**

The systematic review and meta-analysis showed a high HCC incidence and low HBsAg clearance among patients in the IP, especially for HBeAg-negative patients and the Asian population. We emphasize that future multicenter prospective cohort studies or randomized trials are needed to verify if expanding antiviral therapy for patients in the IP is associated with reduced HCC risk or good treatment outcomes.

## Introduction

More than 240 million people worldwide are infected with the chronic hepatitis B (CHB) virus and are at risk for hepatocellular carcinoma (HCC) (Trépo et al., 2014). The natural history of chronic HBV infection includes several defined phases (DP): immune tolerance (IT), HBeAg-positive immune active (IA), inactive hepatitis B surface antigen carriers (IHCs), and HBeAg-negative immune reactivation phase (Gish et al., 2015).

Significantly, emerging data suggest that many CHB patients with increased ALT but low HBV DNA levels, or increased HBV DNA but normal ALT levels, who do not fall into any of the usual immune states are considered to be in the “indeterminate phase (IP)” or “ gray zone” (Hadziyannis, 2007; Yao et al., 2021). It is well accepted within the field that antiviral treatment with either pegylated interferon or a nucleos(t)ide analogue should be offered to CHB patients with liver inflammation and/or moderate-advanced hepatic fibrosis to reduce the progression of liver disease (Yao et al., 2021). The therapeutic goal for new treatments of CHB is to achieve functional cure, that is, hepatitis B surface antigen (HBsAg) clearance (Ning et al., 2019). HBsAg clearance is the desired end point of treatment for CHB patients and is associated with favorable long-term clinical outcomes (Anderson et al., 2021).

Expanding antiviral therapy to benefit more CHB populations and optimizing treatment to reduce the risk of HCC are the two main objectives in current guidelines. However, international guidelines do not recommend antiviral therapy for the treatment of CHB in the IP, mainly due to data being limited on both the risk for HCC and the clinical utility of this indeterminate group. It is unclear whether patients in the IP are at a higher risk for HCC compared to those in the DP and would benefit from antiviral therapy. Therefore, we performed a systematic review and meta-analysis of published literature to evaluate the HCC incidence and HBsAg clearance among patients in IP versus those in DP to better understand this issue.

## Methods

This review was registered in the International Prospective Register of Systematic Reviews (PROSPERO, http://www.crd.york.ac.uk/PROSPERO): CRD42023404002.

### Definitions

We defined the clinical phases as per the AASLD 2018 hepatitis B guidance (**Supplementary Table 1**) (Terrault et al., 2018). Patients were considered to be in the IP when they did not meet the standard criteria for any of the defined phases. IP patients can be divided into four groups: HBeAg-positive, normal ALT, and HBV DNA ≤ 106 IU/ml (IP-A); HBeAg-positive, elevated ALT, and HBV DNA ≤ 20,000 IU/ml (IP-B); HBeAg-negative, normal ALT, and HBV DNA ≥ 2,000 IU/ml (IP-C); and HBeAg-negative, elevated ALT, and HBV DNA ≤ 2,000 IU/ml (IP-D).

### Data sources and search strategy

We followed the Preferred Reporting Items for Systematic Reviews and Meta-Analyses (PRISMA) guidelines (Moher et al., 2015) and searched for studies that reported the incidence of HCC or HBsAg clearance among indeterminate patients. We searched PubMed, Embase, Medline, and Web of Science for articles published in English between 1 January 2007 and 31 March 2023. The entire search strategy is available in **Supplemental File 1**. In addition, we manually searched the bibliography of the included articles and relevant systematic reviews for additional articles. Study authors were contacted directly if necessary for more details.

### Study selection

Randomized controlled trials, and prospective and retrospective cohort studies were eligible for inclusion if they met the following criteria: (a) studies with more than 20 indeterminate adult patients without cirrhosis or HCC at baseline and (b) studies with available adequate data on the primary outcomes, including the frequency and rate of HBsAg clearance (whether spontaneously or after antiviral therapy), or HCC incidence, and an average of more than 1-year of follow-up. There were no geographic restrictions.

Case reports, reviews, guidelines, protocols, fundamental research, letters or comments, and reporting data from duplicate studies were all excluded. If there were multiple studies from the same cohort, we only included data from one representative publication, which provided a complete dataset over the longest time frame. Furthermore, we excluded articles that enrolled patients coinfected with HIV, HCV, and/or HDV to reduce the effects of heterogeneity and other factors on the analysis.

### Data extraction and quality assessment

Two researchers (ML and TXZ) independently screened, assessed the eligibility of studies, and extracted data with a standard form. The senior consulting investigator (JWG) resolved any disagreement on study inclusion or interpretation of data. Data collected included the first author, publication year, region of study, study design, IP group sample size, DP group sample size, sample sizes of treatment, sex, age, and follow-up years. Outcomes included the number of patients with HBsAg clearance and/or HCC incidence, annual HBsAg clearance rate (per 1,000 person-years), and annual HCC incidence (per 1,000 person-years) in different groups (IP group vs. DP group). For articles that did not report the annual HBsAg clearance rate or HCC incidence, we estimated it by using the following formula:


$$\frac{\text{number of patients with HBsAg clearance or HCC incidence}}{\text{mean follow}-\text{up duration}\left(\text{years}\right)\text{ x total number of patients}}\times 1,000$$

Two reviewers (YTZ and AMZ) used the Newcastle–Ottawa assessment scale (NOS) (Stang, 2010) to assess the methodological quality and risk of bias in the nonrandomized studies (**Supplementary Table 2**).

### Statistical analysis

Primary outcomes were the annual HCC incidence and annual HBsAg clearance rates, which were pooled using the random-effects model of DerSimonian and Laird (1986). Because the values of these estimates can be close to 0, we used the Freeman–Tukey Double Arcsine Transformation to stabilize the variance and the Wilson score method to generate 95% confidence intervals (CIs). Heterogeneity across studies was assessed by *I*^2^ statistics and Cochran *Q* test, and a funnel plot and Egger’s test were used to assess any publication bias. Pooled risk ratios (RR) with 95% CIs were calculated to estimate the incidence of HCC and HBsAg clearance rates with the IP group compared with the DP group. All analyses were conducted using the meta package of R software, version 4.3.1. Metarate was used to compute the pooled estimate of both the annual HCC and HBsAg clearance rates, *p*-values were two-tailed, and *p*< 0.05 was considered statistically significant.

## Results

**Figure 1:**
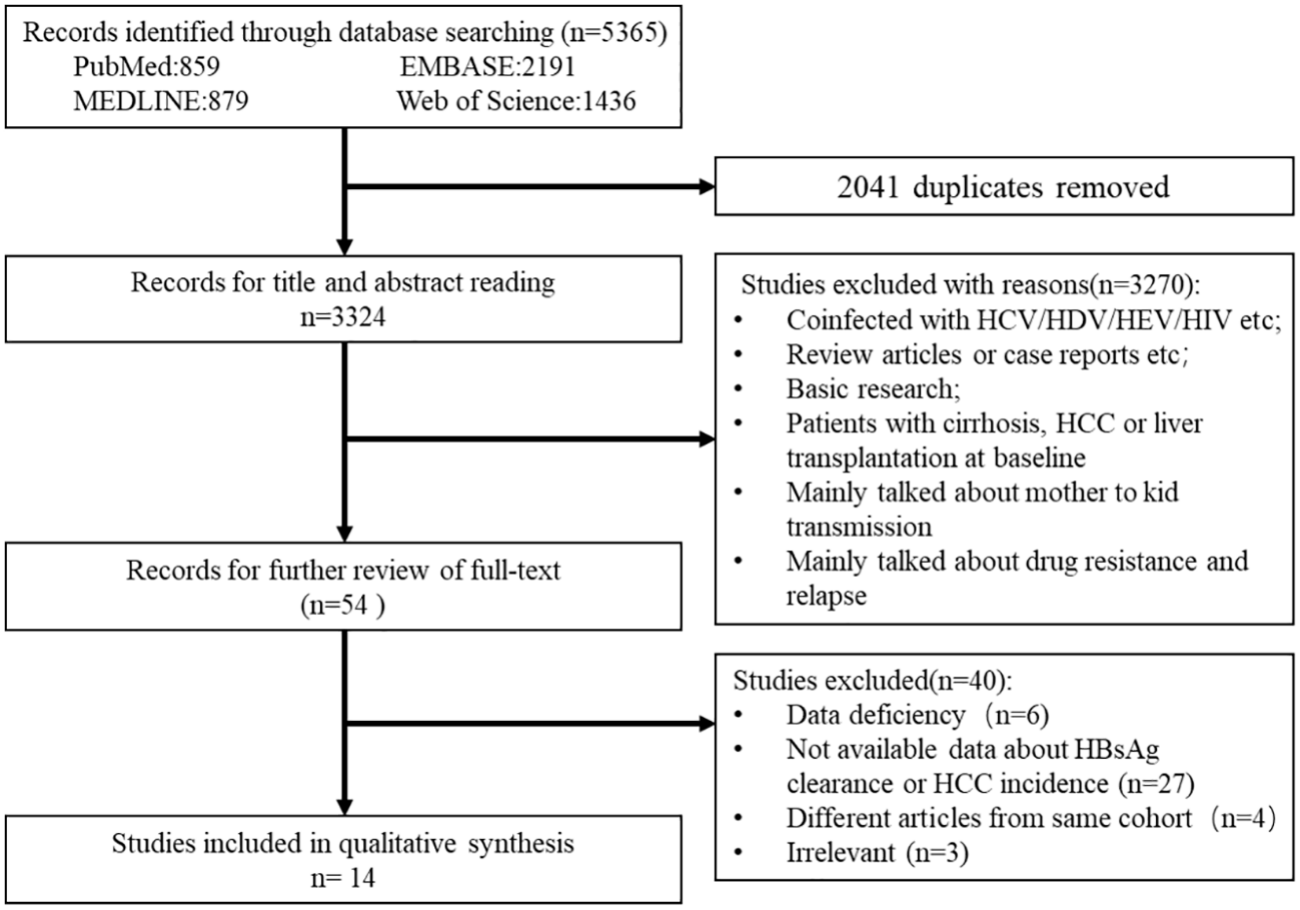
Study selection process.

From the electronic and manual search (**Figure 1**), 3,324 records were identified after removing duplicates and screening the titles and abstracts, and 54 potentially eligible studies were retrieved for full-text reviews. After review, a total of 14 articles (7,798 patients in the IP and 10,725 patients in the DP) were included in the meta-analysis (Yapali et al., 2015; Oliveri et al., 2017; Bonacci et al., 2018; Choi et al., 2019; Lee et al., 2019; Teng et al., 2021; Erken et al., 2022; Huang D. et al., 2022; Huang DQ. et al., 2022; Kim et al., 2022; Koc et al., 2022; Kumada et al., 2022; Tseng et al., 2022; Tseng et al., 2023).

### Characteristics of the studies

**Table 1** shows the characteristics of included studies and of the participants. CHB patients from 14 countries were included, with the majority from the Asia-Pacific region (seven studies). The mean age of the included studies ranged from 31 to 58.2 years, and 9,248 (49.93%) were men.

**Table 1:** Baseline characteristics of included studies.

Study	Region	Design	Sample size		Treat	Male	Age^a^	Follow-up years ^b^	HBsAg clearance				HCC incidence			
									IP		DP		IP		DP	
			IP	DP					Event	Annual incidence (per 1000 P-Y)	Event	Annual incidence (per 1000 P-Y)	Event	Annual incidence (per 1000 P-Y)	Event	Annual incidence (per 1000 P-Y)
Tseng 2023 (Tseng et al., 2023)	Taiwan	RE	1288	1326	No	1585^&^	38.2^&^	12.40^&^	HBeAg+:13/172 HBeAg-:204/1116	HBeAg+:6.1 HBeAg-:14.7	IT:4/125 HBeAg+ IA: 22/206 IHCs:194/822 HBeAg- IA: 28/173	IT:2.6 HBeAg+ IA: 8.6 IHCs:19.0 HBeAg- IA: 13.1	NR	NR	NR	NR
Huang 2022 (Huang D. et al., 2022)	14 centers	RE	819	NR	394	475	46.9	Treat:5.48 Untreat:5.44	NR	NR	NR	NR	Treat:11/394 Untreat:27/425	Treat:5.2 Untreat:11.6	NR	NR
Kim 2022 (Kim et al., 2022)	USA	RE	1093	929	No	NA	49.2*	5.25*	NR	NR	NR	NR	3	0.5	NR	NR
Tsng 2022 (Tseng et al., 2022)	Taiwan	RE	892	1258	No	351* 488 ^#^	42.4^&^	15.88^&^	NR	NR	NR	NR	IP-C:46/658 IP-D: 18/234	IP-C:4.2 IP-D:5.13	IHCs: 16/824 HBeAg- IA: 64/434	IHCs: 1.2 HBeAg- IA: 10.2
Huang 2022 (Huang DQ. et al., 2022)	Taiwan USA	RE	1303	1370	No	898* 966^#^	46.02* 46.46^#^	11.47* 13.67^#^	NR	NR	NR	NR	26/1303	2.6	IHCs:7/1370	IHCs:0.6
Koc 2022 (Koc et al., 2022)	Europe	RE	116	327	No	63* 160 ^#^	31* 32^#^	7* 10^#^	NR	NR	NR	NR	IP-C: 0/116	0	IHCs: 0/327	0
Erken 2022 (Erken et al., 2022)	Europe	PR	46	72	31* 46^#^	69	43^&^	5^&^	IP-C:3/39 IP-D:1/7 Treat:2/31 Untreat: 2/15	IP-C:15.4 IP-D:28.6 Treat:12.9 Untreat: 26.7	IHCs Treat:10/46 Untreat:4/26	IHCs Treat:43.5 Untreat:30.8	0/46	0	0/72	0
Kumada 2022 (Kumada et al., 2022)	Japan	RE	194	332	No	82* 191 ^#^	50* 56^#^	13.4* 14.6^#^	NR	NR	NR	NR	IP-C:3/194	IP-C: 1.2	IHCs: 5/332	IHCs:1.0
Teng 2021 (Teng et al., 2021)	Taiwan	RE	749	NR	292	426	46.5^&^	7.2^&^	NR	NR	NR	NR	Treat:2/292 Untreat:13/457 HBeAg+:5/176 HBeAg-:10/573	Treat: 0.9 Untreat: 3.9 HBeAg+: 3.9 HBeAg-: 2.4	NR	NR
Choi 2019 (Choi et al., 2019)	Korea	RE	900	4118	546^#^	473* 2246^#^	47* 47^#^	7.8* IHCs:9.9 HBeAg-IA:7.9	NR	NR	NR	NR	IP-C:65/900	IP-C: 9.3	IHCs: 140/3572 HBeAg- IA: 57/546	IHCs: 3.9 HBeAg- IA: 13.2
Lee 2019 (Lee et al., 2019)	Korea	RE	152	621	No	81* 345^#^	54.3* 58.2^#^	6.37^&^	NR	NR	NR	NR	HBeA+: 2/39 HBeAg-: 4/113	HBeA+: 8.1 HBeAg-: 5.5	IHCs: 6/621	IHCs: 1.5
Bonacci 2018 (Bonacci et al., 2018)	Spain	RE	114	137	No	71* 80^#^	37* 36^#^	8.2^&^	IP-C: 1/54 IP-D:12/60	IP-C: 2.3 IP-D: 24.4	IHCs:25/137	IHCs: 22.3	IP-C:0/54 IP-D:1/60	IP-C: 0 IP-D: 2.0	IHCs: 0/137	IHCs: 0
Oliveri 2017 (Oliveri et al., 2017)	Italy	PR	46	87	No	27* 49^#^	42.5* 48.0^#^	4.77^&^	IP-C:2/46	IP-C:9.1	IHCs:19/87	IHCs: 45.8	IP-C:0/46	IP-C: 0	IHCs: 0/87	IHCs: 0
Yapali 2015 (Yapali et al., 2015)	USA	RE	86	148	No	122	35^&^	4.25^&^	HBeAg+: 0/4 HBeAg-: 3/82	HBeAg+: 0 HBeAg-: 8.6	IT: 0/24 HBeAg+ IA:0/16 IHCs:6/69 HBeAg- IA: 0/39	IT: 0 HBeAg+ IA: 0 IHCs: 20.5 HBeAg- IA: 0	HBeAg+: 0/4 HBeAg-:1/82	HBeAg+: 0 HBeAg-: 2.9	IT: 0/24 HBeAg+ IA: 0/16 IHCs: 0/69 HBeAg- IA: 1/39	IT: 0 HBeAg+ IA: 0 IHCs: 0 HBeAg- IA: 6.0

IP, indeterminate phase; DP, defined phases; NR, Not Reference; RE, Retrospective; PR, Prospective; IT, immune tolerance; IHCs, inactive hepatitis B surface antigen carriers; IA, immune active; P-Y, Person-Year.

* for the IP group, # for the None-IP group;&Data from the whole cohort.

a Age was presented as mean, median;

b Follow-up year was presented as means or median.

### The annual HCC incidence and subgroup analysis in the IP patients

**Figure 2:**
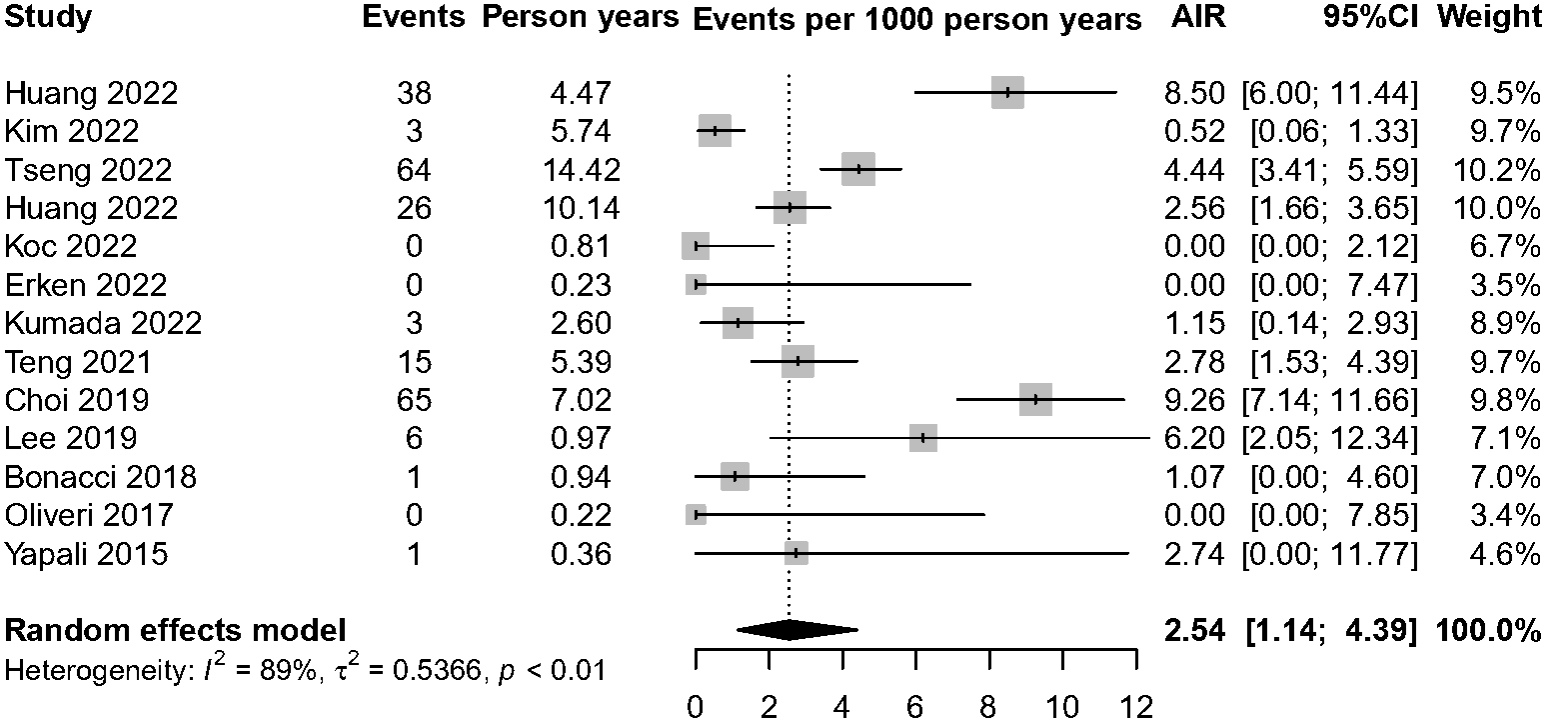
Annual HCC incidence rate among IP patients. AIR, annual incidence rate.

**Figure 3:**
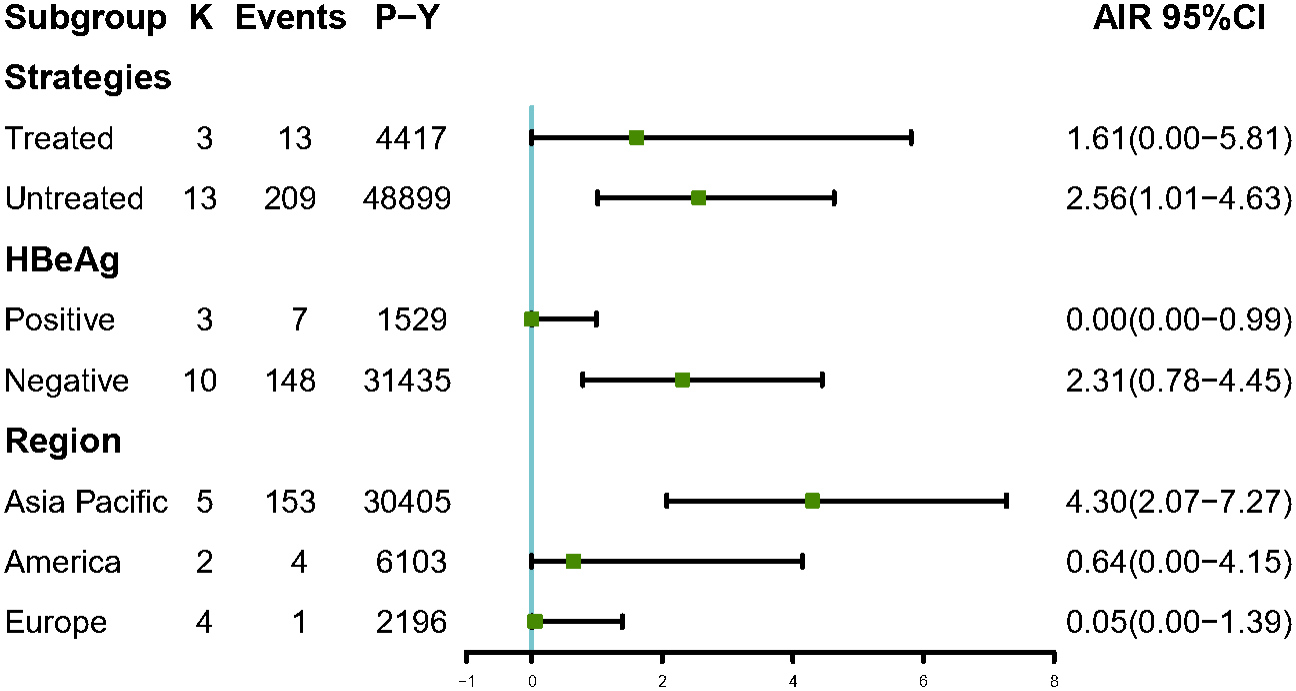
Subgroup analysis of annual HCC incidence by strategies, HBeAg status and region among IP patients. AIR, Annual incidence rate. K, number of studies; P-Y, person-years.

Among 6,510 patients in the IP, 222 patients developed HCC. The annual HCC rates ranged from 0% to 9.26%, with a pooled incidence of 2.54 cases per 1,000 person-years (95% CI, 1.14–4.39, *I*2 = 89%, random-effects model, **Figure 2**), and no publication bias as assessed by visual inspection of the funnel plots (**Supplementary Figure 1**) and results of Egger’s test (*p* = 0.504). To minimize data heterogeneity among studies, we further performed a subgroup analysis. The pooled annual HCC incidence rates were higher among untreated patients (2.56%, 95% CI, 1.01–4.63) than treated patients (1.61%, 95% CI, 0.00–5.81), although the difference was not statistically significant (*p* = 0.09) (**Supplementary Figure 2**). We further analyzed HCC incidence by different HBeAg status and found that HBeAg-negative patients (2.31%; 95% CI, 0.87–4.45) showed higher pooled annual rates of HCC incidence than HBeAg-positive patients (0.00%; 95% CI, 0.00–0.99) (*p*< 0.001). In addition, we stratified by geographic region, and the results showed Asia-Pacific region (4.30%; 95% CI, 2.07–7.27) also had significantly higher annual rates than Europe (0.05%; 95% CI, 0.00–1.39) (*p*< 0.001)), but there was no significant difference between Asia-Pacific and America (0.64%; 95% CI, 0.00–4.15) (*p* = 0.22) (**Figure 3**).

### Incidence of HCC among CHB patients in the IP versus DP

**Figure 4:**
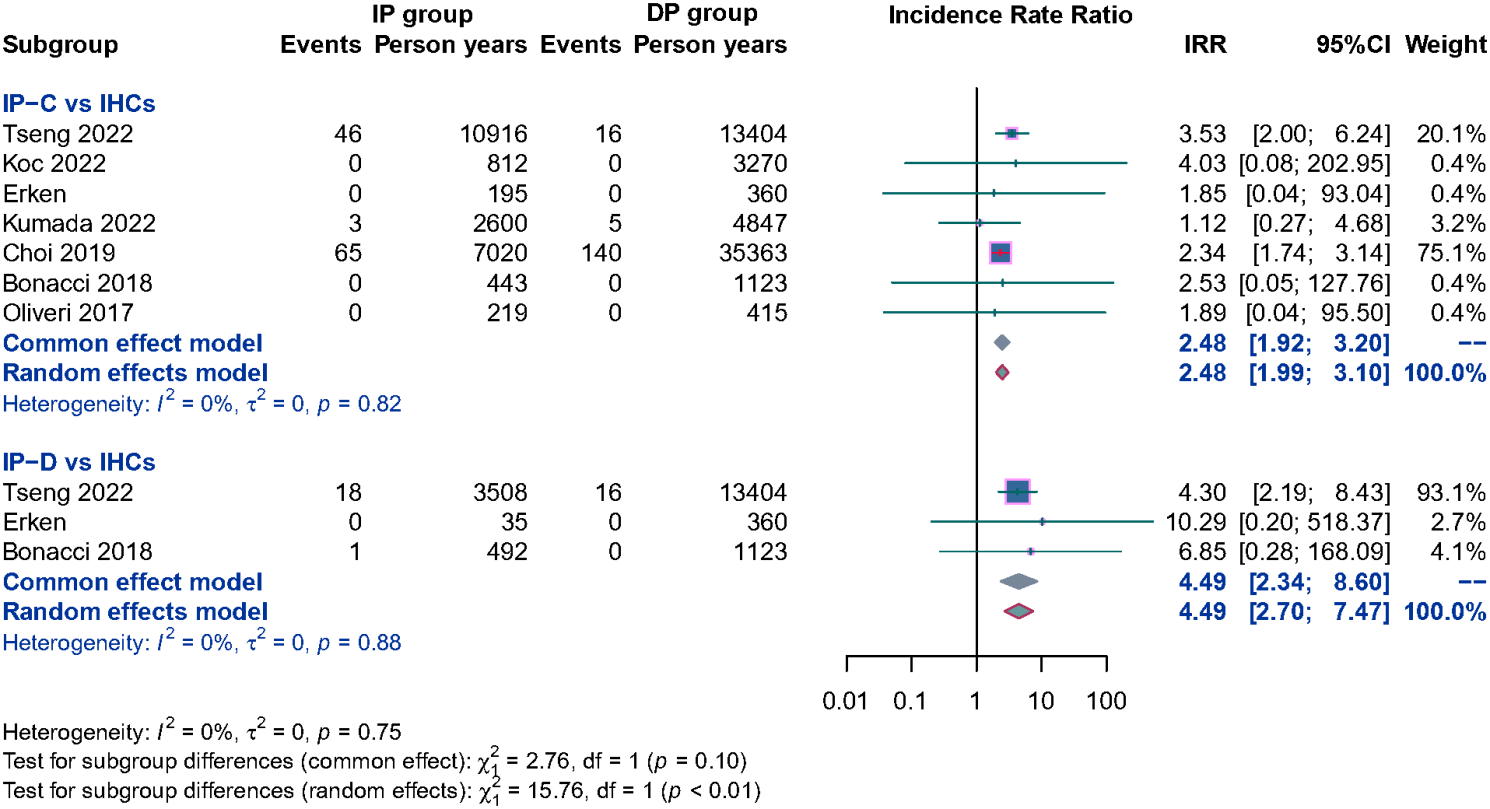
Subgroup analysis of annual HCC incidence among CHB patients in the HBeAg negative IP (IP-C and IP-D) versus IHCs.

Ten studies separately reported the HCC incidence among CHB patients in the IP (*n* = 3,849) versus the DP (*n* = 8,470), and the result showed that patients in the IP group were associated with a significantly higher HCC incidence than DP patients; the pooled RR is 1.64 (95% CI, 1.34–2.00, *p*< 0.001) (**Supplementary Figure 3**). To evaluate the potential influence of different natural history, subgroup analyses were performed. IP-C patients had a 2.48 times higher annual HCC incidence rate than IHC patients. Similarly, IP-D patients also had significantly higher HCC incidence than IHC patients, and the pooled RR was 4.49 (95% CI, 2.70–7.47, *p*< 0.001) (**Figure 4**). Because of the limited number of relevant studies, we did not conduct a subgroup analysis comparing HBeAg-positive IP patients with DP patients.

### The annual HBsAg clearance among CHB patients in the IP versus DP

**Figure 5:**
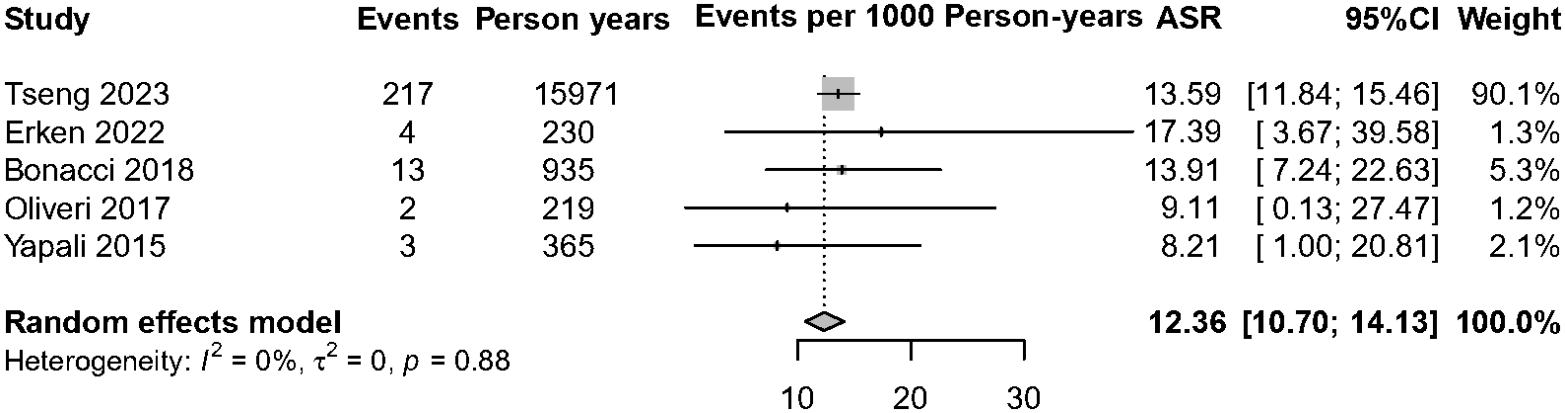
Annual HBsAg clearance rate among IP patients. ASR, annual seroclearance rate.

Currently, the existing literature is sparse on HBsAg clearance of IP patients. Among the five studies with 1,580 IP patients on HBsAg clearance, over 98% of patients (*n* = 1,549) have no antiviral treatment. A total of 239 patients achieved HBsAg clearance, and the annual HBsAg clearance pooled incidence was 12.36 cases per 1,000 person-years (95% CI, 10.70–14.13, *I*^2 =^ 0%, common-effect model, **Figure 5**). We further analyzed the data of patients who achieved HBsAg clearance in IP versus DP patients (*n* = 1,770). The result showed that IP patients were associated with a lower annual HBsAg clearance rate compared with the DP patients (RR = 0.83, 95% CI, 0.70–0.99, *p* = 0.03) (**Supplementary Figure 4**).

## Discussion

In this meta-analysis of 14 studies, which included 7,798 IP patients, a total of 222 patients developed HCC, and 239 patients achieved HBsAg clearance, resulting in a pooled annual HCC incidence of 2.54 cases per 1,000 person-years (95% CI, 1.14–4.39) and HBsAg clearance rates of 12.36 cases per 1000 person-years (95% CI, 10.70–14.13). In addition, we found that IP patients were associated with significantly higher HCC incidence risk (RR = 1.64, 95% CI, 1.34–2.00) and slightly lower annual HBsAg clearance rates (RR = 0.83, 95% CI, 0.70–0.99) than the DP patients; expanding antiviral therapy might be beneficial in patients in this phase.

The HCC pooled annual incidence rates ranged from 0% to 9.26% across the studies due to different strategies (antiviral treatment or observation), HBeAg status, or regions. By subgroup analyses, we found that IP patients who were HBeAg negative at baseline from the Asia-Pacific region had a higher risk of developed HCC. The reasons may partly be because HBV infection often occurs in young people, and patients usually have a long disease course by the CHB stage in Asian populations. Another possible reason is the differences in the geographic distribution of HBV genotypes. Genotype A is most frequent in North America and Africa, genotypes B and C are the dominant viruses throughout East Asia, and genotype D is most common in Southern Europe and India (Tong and Revill, 2016). Analysis of patients by HBV genotypes was not performed because of a lack of primary data, with most included studies not reporting HBV genotype information. Notably, a retrospective study by Huang et al. (2023) showed that antiviral therapy reduces HCC risk by 70% among IP CHB patients. In this meta-analysis, the pooled annual HCC incidence rates were higher among untreated patients (2.56%, 95% CI, 1.01–4.63) than treated ones (1.61%, 95% CI, 0.00–5.81), but the difference was not statistically significant (*p* = 0.09). However, the severe heterogeneity of our results requires that they be interpreted with caution.

Furthermore, we found that IP patients, especially IP-C and IP-D patients, were associated with significantly higher HCC incidence risk than the IHCs who were HBeAg negative, had lower HBV DNA levels, and had quantitative HBsAg (Cao et al., 2017). In addition, owing to the limited number of relevant studies, we did not conduct a subgroup analysis comparing HBeAg-positive IP patients versus DP patients.

CHB patients spontaneously achieving HBsAg clearance is a rare event. Of note, over 98% of IP patients had no antiviral treatment among the included five studies in this meta-analysis, a total of 237 patients spontaneously achieved HBsAg clearance among 1,580 IP patients, the annual HBsAg clearance pooled incidence was 12.36 cases per 1,000 person-years (95% CI, 10.70–14.13), and a high rate of HBsAg spontaneous clearance was observed in IP patients, probably because 88.86% of patients (1,404/1,580) were HBeAg negative at baseline, which was associated with a high rate of HBsAg clearance. Nevertheless, the pooled RR was 0.89 among CHB patients in the IP versus DP; the differences still reached statistical significance (*p* = 0.03).

This study has some limitations. First, there are different definitions of normal ALT among different international guidelines. The ALT cutoff is 35 U/L for male individuals and 25 U/L for female individuals defined by the AASLD 2018 guideline (Terrault et al., 2018); however, the upper limit of normal ALT is 40 U/L based on EASL and APASL guidelines (Sarin et al., 2016; Lampertico et al., 2017). In general, the IP was defined according to the AASLD 2018 guidance in this meta-analysis, but because the existing literature lacks relevant IP studies, we did not strictly follow the criteria of normal ALT defined by the AASLD 2018 guideline. Second, owing to the limited number of relevant HBeAg-positive studies and the fact that the sample sizes in available studies are usually small, the results of this meta-analysis mainly reflect in HBeAg-negative IP patients of HCC incidence/HBsAg clearance. Third, because current international guidelines do not recommend treatment for IP patients, few prospective cohort studies or RCT studies have been conducted to investigate post-treatment HBsAg clearance and long-term HCC incidence. Fourth, some subgroup analyses in our study included a relatively small number of studies and subjects, causing inaccurate estimation. Therefore, the results of this meta-analysis have some inherent heterogeneities.

To the best of our knowledge, this is the first systematic study with a large sample size to analyze the annual HCC incidence and HBsAg clearance rates for CHB patients in the IP. In short, this meta-analysis shows that IP patients were associated with significantly higher HCC incidence risk and slightly lower annual HBsAg clearance rates than the DP patients. We emphasize that future multicenter prospective cohort studies or randomized trials are needed to verify if expanding antiviral therapy for patients in the IP is associated with reduced HCC risk or good treatment outcomes.

## Author contributions

ML, JG, and XX conceived and designed the protocol and study. ML and TZ identified studies to be screened. YZ and A-MZ identified studies for eligibility, extracted data, and assessed the methodological quality of the included studies. ML performed the analysis with assistance from TZ and YZ. All authors contributed to the article and approved the submitted version.
