# Supplementary material for: The incidence of hepatocellular carcinoma and clearance of hepatitis B surface for CHB patients in the indeterminate phase: a systematic review and meta-analysis
Source: Front Cell Infect Microbiol. 2023 Sep 12;13:1226755. doi: 10.3389/fcimb.2023.1226755 (PMC10523783; doi:10.3389/fcimb.2023.1226755)
Supplement: Supplementary file 1 [file DataSheet_1.docx]

**Supplementary Materials**

**Supplemental file 1. Searching strategy and result.**

**Number of citations by each database searched**

| **Databases** | **Citations** |
| --- | --- |
| PubMed | 859 |
| EMBASE | 2191 |
| MEDLINE | 879 |
| Web of Science | 1436 |
| Total databases | 5365(with 2041 duplicates) |
| Total databases after removing duplicates | 3324 |

Searching strategy for PubMed

1. "Hepatitis B, Chronic"[Mesh]
2. "Chronic Hepatitis B" [Title/Abstract]
3. "Chronic HBV infection" [Title/Abstract]
4. CHB [Title/Abstract]
5. 1 OR 2 OR 3 OR 4
6. "Alanine Transaminase"[Mesh]
7. "Alanine aminotransferase" [Title/Abstract]
8. ALT [Title/Abstract]
9. Grey zone [Title/Abstract]
10. Indeterminate [Title/Abstract]
11. 6 OR 7 OR 8 OR 9 OR 10
12. "Hepatitis B Surface Antigens" [Mesh]
13. HBsAg [Title/Abstract]
14. 12 OR 13
15. seroclearance [Title/Abstract]
16. loss [Title/Abstract]
17. seroconversion [Title/Abstract]
18. clearance [Title/Abstract]
19. 15 OR 16 OR 17 OR 18
20. 14 AND 19
21. "Carcinoma, Hepatocellular" [Mesh]
22. "Hepatocellular Carcinoma" [Title/Abstract]
23. HCC [Title/Abstract]
24. clinical outcome [Title/Abstract]
25. advanced liver disease [Title/Abstract]
26. 21 OR 22 OR 23 OR 24 OR 25
27. 20 OR 26
28. 5 AND 11 AND 27 AND Filter: humans, English, from **2007/1/1 - 2023/3/31**

**Search strategy for Embase**

1. 'Hepatitis B, Chronic'/exp/mj
2. 'Chronic Hepatitis B':ab,ti
3. 'Chronic HBV infection ':ab,ti
4. 'CHB':ab,ti
5. 1 OR 2 OR 3 OR 4
6. 'Alanine Transaminase'/exp/mj
7. 'Alanine aminotransferase':ab,ti
8. 'ALT':ab,ti
9. 'Grey zone':ab,ti
10. 'Indeterminate':ab,ti
11. 6 OR 7 OR 8 OR 9 OR 10
12. 'Hepatitis B Surface Antigens'/exp/mj
13. 'HBsAg':ab,ti
14. 12 OR 13
15. 'seroclearance':ab,ti
16. 'loss':ab,ti
17. 'seroconversion':ab,ti
18. 'clearance':ab,ti
19. 15 OR 16 OR 17 OR 18
20. 14 AND 19
21. 'Carcinoma, Hepatocellular'/exp/mj
22. 'Hepatocellular Carcinoma':ab,ti
23. 'HCC':ab,ti
24. 'clinical outcome':ab,ti
25. 'advanced liver disease':ab,ti
26. 21 OR 22 OR 23 OR 24 OR 25
27. 20 OR 26
28. #5 AND #11 AND #27 AND [english]/lim AND [humans]/lim AND [embase]/lim AND [2007-2023]/py

**Search strategy for MEDLINE and Web of Science (similar to pubmed)**

**Supplemental Table 1: Indeterminate Phase Defined by American Association for the Study of liver Disease 2018 Criteria**

|  | **HBeAg** | **HBV DNA**  **(IU/mL)** | **ALT(U/L)** | **Liver biopsy** |
| --- | --- | --- | --- | --- |
| **Immune tolerant** | postive | ＞106 | ＜1xULN | no fibrosis and minimal inflammation |
| **Immune active** | postive | ≥20000 | ≥2xULN | moderate or severe necroinflammation and with or without fibrosis |
|  | negative | ≥2000 | ≥2xULN |  |
| **Inactive** | negative | ＜2000 | ＜1xULN | absence of significant necroinflammation, variable levels of fibrosis |
| **Indeterminate** | **CHB patients who does not fit the above criteria** | | | |

**ULN, upper limit of normal (35 U/L for males, 25 U/L for females)**

**Supplemental Table 2: Quality assessment of cohort studies**

|  | **Selection** | | | | **Comparability** | **Outcome** | | | **Total score** |
| --- | --- | --- | --- | --- | --- | --- | --- | --- | --- |
| **Author, year** | **Representativeness of the exposed cohort** | **Selection of the non-exposed cohort** | **Ascertainment of exposure** | **Outcome not present at start of study** | **Comparability of cohorts on the basis of the design or analysis** | **Assessment of outcome** | **Follow-up long enough for outcomes to occur** | **Adequacy of follow-up of cohorts** |  |
| Tseng et al, 2023 | A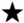 | B | A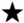 | A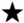 | A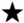 | B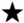 | A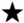 | A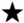 | 7 |
| Huang et al, 2022 | A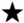 | A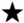 | A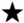 | A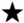 | A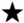 | B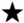 | A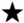 | B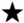 | 8 |
| Kim et al, 2022 | A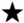 | B | A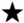 | A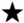 | A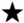 | B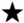 | A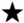 | D | 6 |
| Tseng et al, 2022 | A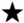 | B | A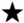 | A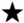 | A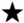 | A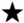 | A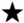 | A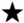 | 7 |
| Huang et al, 2022 | A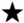 | B | A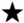 | A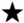 | A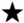 B 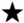 | A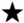 | A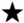 | A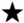 | 8 |
| Koc et al, 2022 | A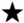 | B | A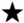 | A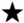 | A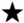 | A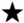 | A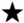 | A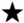 | 7 |
| Erken et al, 2022 | B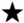 | B | A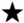 | A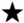 | A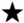 | A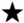 | A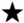 | B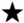 | 7 |
| Kumada et al, 2022 | A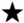 | B | A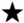 | A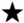 | A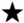 | A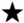 | A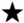 | A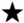 | 7 |
| Teng et al, 2021 | A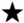 | A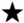 | A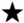 | A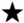 | A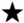 | A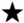 | A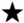 | B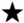 | 8 |
| Choi et al, 2019 | A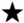 | B | A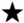 | A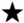 | A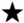 | A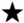 | A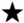 | A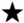 | 7 |
| Lee et al, 2019 | A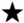 | B | A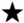 | A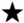 | A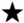 | A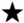 | A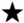 | A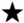 | 7 |
| Bonacci et al, 2018 | A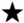 | B | A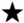 | A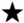 | A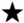 | A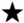 | A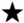 | A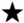 | 7 |
| Oliveri et al, 2017 | A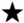 | B | A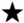 | A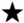 | A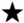 | A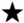 | A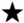 | A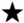 | 7 |
| Yapali et al, 2015 | A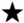 | B | A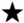 | A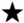 | A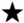 | A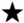 | A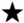 | A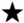 | 7 |

| **Figure S1. Funnel plot of pooled annual HCC incidence rate meta-analysis among IP group.** |
| --- |

| **Figure S2. Subgroup Meta-analysis of the pooled annual HCC incidence rate among IP patients** |
| --- |

| **Figure S3. Meta-analysis of the pooled annual HCC incidence among IP group versus DP group** |
| --- |

| **Figure S4. Meta-analysis of the pooled annual HBsAg clearance rate among IP group versus DP group** |
| --- |
